# The impact of early administration of vasopressor agents for the resuscitation of severe hemorrhagic shock following blunt trauma

**DOI:** 10.1186/s12873-020-00322-1

**Published:** 2020-04-16

**Authors:** Kenichiro Uchida, Tetsuro Nishimura, Naohiro Hagawa, Shinichiro Kaga, Tomohiro Noda, Naoki Shinyama, Hiromasa Yamamoto, Yasumitsu Mizobata

**Affiliations:** Department of Traumatology and Critical Care Medicine, Osaka City University, Graduate school of medicine, , 1-5-7, Asahi-machi, Abeno-ku, Osaka City, Osaka 545-8585 Japan

**Keywords:** Resuscitation, Vasopressor, Hemorrhagic shock, Blunt trauma, Multiple trauma

## Abstract

**Background:**

When resuscitating patients with hemorrhagic shock following trauma, fluid volume restriction and permissive hypotension prior to bleeding control are emphasized along with the good outcome especially for penetrating trauma patients. However, evidence that these concepts apply well to the management of blunt trauma is lacking, and their use in blunt trauma remains controversial. This study aimed to assess the impact of vasopressor use in patients with blunt trauma in severe hemorrhagic shock.

**Methods:**

In this single-center retrospective study, we reviewed records of blunt trauma patients with hemorrhagic shock and included patients with a probability of survival < 0.6. Vital signs on arrival, characteristics, examinations, concomitant injuries and severity, vasopressor use and dose, and volumes of crystalloids and blood infused were compared between survivors and non-survivors. Data are described as median (25–75% interquartile range) or number.

**Results:**

Forty patients admitted from April 2014 to September 2019 were included. Median Injury Severity Score in survivors vs non-survivors was 41 (36–48) vs 45 (34–51) (*p* = 0.48), with no significant difference in probability of survival between the two groups (0.22 [0.12–0.48] vs 0.21 [0.08–0.46]; *p* = 0.93). Despite no significant difference in patient characteristics and injury severity, non-survivors were administered vasopressors significantly earlier after admission and at significantly higher doses. Total blood transfusion amount administered within 24 h after admission was significantly higher in survivors (8430 [5680–9320] vs 6540 [4550–7880] mL; *p* = 0.03). Max catecholamine index was significantly higher in non-survivors (2 [0–4] vs 14 [10–18]; *p* = 0.008), and administered vasopressors were terminated significantly earlier (12 [4–26] vs 34 [10–74] hours; *p* = 0.026) in survivors.

Although the variables of severity of the patients had no significant differences, vasopressor use (Odds ratio [OR] = 21.32, 95% confident interval [CI]: 3.71–121.6; *p* = 0.0001) and its early administration (OR = 10.56, 95%CI: 1.90–58.5; *p* = 0.005) indicated significant higher risk of death in this study.

**Conclusion:**

Vasopressor administration and high-dose use for resuscitation of hemorrhagic shock following severe blunt trauma are potentially associated with increased mortality. Although the transfused volume of blood products tends to be increased when resuscitating these patients, early termination of vasopressor had better to be considered.

## Background

In the resuscitation of hemorrhagic shock, the priority in its management is the absolute and immediate control of sites of bleeding along with simultaneous volume resuscitation to maintain adequate tissue perfusion [1]. Historically, the importance of permissive hypotension and the restriction of crystalloid fluid volume before hemorrhage control has been emphasized with good outcomes in patients with penetrating trauma [2, 3], but recently, several studies have indicated uncertainness of the use of permissive hypotension in trauma patients [4–6]. Furthermore, there is still no evidence of these concepts being successfully applied to the management of patients following blunt injury or those with traumatic brain injury (TBI) [7–9].

Despite these controversies, vasopressors are still globally administered in some trauma patients in severe shock to maintain minimal perfusion pressure especially for the brain or are sometimes used as fluid-sparing adjuncts to resuscitation without diluting clotting factors [10, 11]. Although many reports do not recommend the use of vasopressors for the resuscitation of trauma patients [12, 13], some reports and guidelines have committed to the temporary use of vasopressors for life-threatening hemorrhagic shock to minimize fluid volume administered and maintain appropriate systemic perfusion [14, 15]. Even in level 1 trauma centers, where any surgical or interventional radiographic procedures for the immediate control of bleeding and early activation of massive transfusion protocol (MTP) are always available, the effects and risks of vasopressor administration for severe hemorrhagic shock following trauma remain unclear [16–19].

Thus, the objective of this study was to evaluate the impact of vasopressor use in patients with blunt traumatic injury who are in severe hemorrhagic shock.

## Methods

### Patient selection

This was a single-center retrospective review of patients admitted to the Trauma and Critical Care Center of Osaka City University Hospital, a level 1 urban area trauma center in the second largest city by population in Japan. We reviewed all patients admitted with trauma during April 2014 and September 2019 who were over 16 years old and included those patients with hemorrhagic shock following blunt trauma injury to the torso and required immediate intervention such as surgery or trans-arterial embolization. In the current study, hemorrhagic shock was defined as systolic blood pressure less than 100 mmHg on arrival or a ratio of heart rate over systolic blood pressure of > 1 and an increased lactate level of > 2.5 mmol/L.

We hypothesized that patients with mild to moderate injury simply tend not to be administered vasopressor agents and if administrated, the outcomes of these patients would not be affected by the use of these agents. Under this hypothesis, to evaluate exact impacts of vasopressor administration in severe trauma patients, we excluded the patients with a probability of survival (Ps) score calculated by the Trauma and Injury Severity Score (TRISS) [20, 21] of ≥0.6. To evaluate the outcomes and complications of vasopressor administration for the patients, we also excluded patients in cardiopulmonary arrest on arrival, those dying for a brief instant of time within 24 h after arrival, and patients transferred from other hospitals. The patients were divided into survivors who conclusively discharged to home or transferred for rehabilitation or non-survivors who died during admission.

### Data collection

The factors which supposed to influence the outcomes were investigated from the clinical records. Patient’s demographics such as sex, age, past medical history, mechanism of injury, injury severity score and physiological data on arrival were compared.

We also compare the examination results like the results of focused assessment with sonography for trauma (FAST), blood gas data on admission, concomitant injuries, clinical time flow and procedures, total amount of blood transfusion, external cellular fluid in addition to the vasopressor administration and it’s timing. These data were all recorded without deficit in our institutional registry.

### Resuscitative strategy for trauma and Administration of Vasopressor Agents

Our resuscitation strategy for trauma patients in shock is based on the Advanced Trauma Life Support guidelines and basically emphasizing the importance of early administration of blood transfusion. When a trauma patient arrives at the hospital, we initially start fluid resuscitation with extracellular fluid infusion. If the patient has sites of bleeding, we assessed the response of hemodynamics to extracellular fluid resuscitation of < 1000 mL. Then, if the hemodynamic condition of the patient is still unstable, we immediately administer a transfusion of blood. Although we stock 20 units of red blood cells and 20 units of fresh frozen plasma in the resuscitation room, MTP including cryoprecipitate is always available. Our MTP is based on the current 1:1:1 ratio theory of usage of platelets:fresh frozen plasma:red blood cells, and packs are constantly brought to the resuscitation room with that ratio of composition.

As the highest priority for hemorrhage is control of the sites of bleeding, if the patient is not stable enough to transfer to the operating room, we perform abbreviated interventions such as emergent resuscitative thoracotomy, laparotomy, or retroperitoneal packing in the resuscitation room if needed.

The indications for the administration and timing of vasopressor agents in the resuscitation are completely up to the preference of the trauma surgeon or physician leading the resuscitation. When vasopressors are used in conjunction with volume resuscitation, we usually administer norepinephrine first, especially for hemorrhagic shock, but if a low cardiac ejection fraction is quickly estimated from wall motion in the initial FAST, dopamine is used first in some patients. We defined the early administration of vasopressor as either dopamine or noradrenaline started within 1 h after admission.

### Statistical analysis

All statistical data are presented as the median (25–75% interquartile range [IQR]) or number. Univariate comparisons between the survivor and non-survivor were performed for the factors of demographic, blood examinations, injury severities, and clinical courses. Categorical variables were analyzed with Fischer’s exact test. Non-parametric numerical data (presented as median with IQR) were compared using the Mann-Whitney *U*-test.

Multiple logistic regression analysis was performed for identifying factors affecting survival. The factors significantly different between the two groups in the univariate comparison were used for the independent variables to identify the affecting factors for survival in the multiple logistic regression analysis. A value of *p* < 0.05 was considered statistically significant. Data were analyzed using IBM SPSS Statistics, version 22 (SPSS Inc., Chicago, IL).

## Results

In total, 318 patients were admitted during the study period with hemorrhage following traumatic torso injury and required immediate intervention to control bleeding. Ninety-two patients were excluded because of cardiopulmonary arrest on arrival or death within 24 h after arrival, as were 52 patients with penetrating injury. As six patients were transferred from the other hospitals and 128 patients were of more than 0.6 of Ps, these patients were also excluded.

**Fig. 1:**
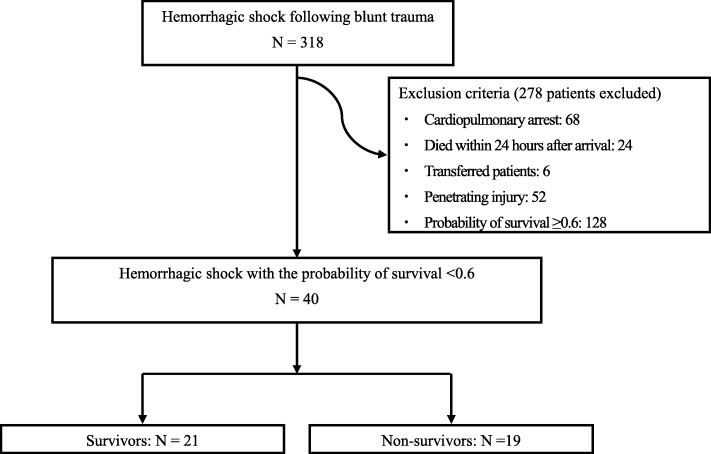
Patient selection

Finally, 40 patients with blunt trauma and a Ps < 0.6 were included, and their clinical results were compared in this study (Fig. 1).

The epidemiologic characteristics and initial clinical presentation of the patients in the survivor and non-survivor groups are shown in Table 1. The median age of the survivors vs non-survivors was 58 (42–68) vs 62 (45–73) years old (*p* = 0.09), and almost 60% of patients were males in both groups. There were no significant differences between the two groups in the mechanism of injury. The Injury Severity Score (ISS) was 41 (36–48) vs 45 (34–51) (*p* = 0.48), respectively. There were no significant differences in physiological signs such as Glasgow Coma Scale, heart rate, respiratory rate, body temperature and systolic blood pressure between the two groups. The Ps score between the two groups also was not significantly different (0.22 [0.12–0.48] vs 0.21 [0.08–0.46]; *p* = 0.93).

**Table 1:** Demographic data of the patients

	*Survivors* *N = 21*	*Non-survivors* *N = 19*	*p value*
*Sex, male/female*	*12/ 9 (Male 57.1%)*	*10/ 9 (Male 52.6%)*	*1.00*
Age, years	58 (42–68)	62 (45–73)	0.09
ISS	41 (36–48)	45 (34–51)	0.48
Mechanism of injury			0.53
Motor vehicle accident	9 (42.9%)	11 (57.9%)	
Fall from height	12 (57.1%)	8 (42.1%)	
Physiological data on arrival			
GCS	8 (4–13)	6 (3–12)	0.17
RR (breaths per min)	28 (24–30)	30 (20–33)	0.88
HR (beats per min)	120 (112–124)	112 (98–120)	0.07
Systolic blood pressure (mmHg)	90 (64–104)	76 (40–95)	0.25
Body temperature (°C)	36.1 (35.7–36.5)	35.8 (34.9–36.2)	0.11
Past medical history			
Stroke	2 (9.5%)	1 (5.3%)	1.0
Cardiac failure	3 (14.3%)	1 (5.3%)	0.6
Respiratory disease	3 (14.3%)	2 (10.5%)	1.0
Chronic kidney disease	2 (9.5%)	0	0.49
Probability of survival	0.22 (0.12–0.48)	0.21 (0.08–0.46)	0.93

*ISS* injury severity score, *GCS* Glasgow coma scale, *RR* respiratory rate, *HR* heart rate

Statistical data are presented as median (25–75% IQR) or number

Table 2 shows the results of examinations on admission. There were no significant differences between the two groups with regard to the results of extended FAST, base excess, pH and lactate level. None of the coagulation-related factors showed a significant difference between the survivors and non-survivors. The injury descriptions classified according to location are shown in Table 3 and we found no significant differences between the two groups.

**Table 2:** Examination results

	*Survivors* *N = 21*	*Non-survivors* *N = 19*	*p value*
*Positive extended FAST*	*14* (66.7%)	*10* (52.6%)	*0.52*
Base excess	−8.6 (− 12.1--4.8)	−9.6 (− 13.2--5.1)	0.42
pH	7.24 (7.15–7.37)	7.20 (7.13–7.7.31)	0.53
Lactate level (mmol/L)	6.8 (3.8–7.8)	8.8 (4.7–10.4)	0.15
Fibrinogen level (mg/dL)	170 (169–236)	167 (144–255)	0.38
PT-INR	1.12 (1.01–1.15)	1.22 (1.05–1.28)	0.36

*FAST* focused assessment with sonography for trauma, *PT-INR* prothrombin time-international normalized ratio

Statistical data are presented as median (25–75% IQR)

**Table 3:** Injuries occurring in the patients

	*Survivors* *N = 21*	*Non-survivors* *N = 19*	*p value*
Injury			
Cranial injury (GCS > 8)	8 (38.1%)	7 (36.8%)	1.00
Craniofacial injury	4 (19.0%)	3 (16.8%)	1.00
Thoracic injury	16 (76.2%)	14 (73.7%)	1.00
Abdominal injury	14 (66.7%)	9 (47.4%)	0.34
Pelvic injury	8 (38.1%)	11 (57.9%)	0.34
Bony spinal injury	5 (23.8%)	8 (42.1%)	0.31

*GCS* Glasgow coma scale

Statistical data are presented as median (25–75% IQR) or number

The clinical courses of the patients are shown in Table 4. MTP was activated in all patients and 6 of 21 (28.6%) vs 8 of 19 (42.1%) patients (*p* = 0.51) underwent aortic cross- clamping including resuscitative endovascular balloon occlusion of the aorta (REBOA) in each group. Aortic clamping was performed at a significantly higher rate in the non-survivors. The median time from presentation to the hospital to start surgical procedures for stop the bleeding was 32 (15–43) vs 38 (18–48) minutes (*p* = 0.67) and that of interventional radiology was 62 (54–76) vs 67 (59–78) minutes (*p* = 0.79). There were no significant differences in the volume of external cellular fluid infused in the resuscitation room before the administration of blood transfusion (750 [400–900] vs 800 [450–960] mL (*p* = 0.48). The total amount of blood transfused within 24 h after admission was significantly higher in the survivors (8430 [5680–9320] vs 6540 [4550–7880] mL; *p* = 0.03). Vasopressors were also administered significantly earlier and at higher doses in the non-survivors compared with the survivors. The patients who were administered a vasopressor within one hour after admission had significantly high mortality. The score of the max catecholamine index calculated as [noradrenaline * 100 + dopamine γ] was significantly higher in the non-survivors (2 [0–4] vs 14 [10–18]; *p* = 0.008). The administered vasopressors were terminated significantly earlier in the survivors compared with the non-survivors (12 [4–26] vs 34 [10–74] hours; *p* = 0.026).

**Table 4:** Clinical courses

	*Survivors* *N = 21*	*Non-survivors* *N = 19*	*p value*
Activation of MTP	21 (100%)	19 (100%)	1.00
Aortic cross-clamping including REBOA	6 (28.6%)	8 (42.1%)	0.51
Infused volume of ECF (mL)	750 (400–900)	800 (450–960)	0.48
Time to intervention (min)			
Surgery	32 (15–43)	38 (18–48)	0.67
Interventional radiology	62 (54–76)	67 (59–78)	0.79
Total amount of blood transfusion (mL)	8430 (5680–9320)	6540 (4550–7880)	0.03
Vasopressor use	6 (28.6%)	17 (89.5%)	0.0001
Max catecholamine index	2 (0–4)	14 (10–18)	0.008
Vasopressor use < 1 h after admission	2 (9.5%)	9 (47.4%)	0.001
Time to vasopressor termination (h)	12 (4–26)	34 (10–74)	0.026

*MTP* massive transfusion protocol, *REBOA* resuscitative endovascular balloon occlusion of the aorta, *ECF* external cellular fluid

Catecholamine index = [noradrenaline * 100 + dopamine]

Statistical data are presented as median (25–75% IQR) or number

Although the variables of aortic clamp seemed no significant risk factors for mortality, vasopressor use (Odds ratio [OR] = 21.32, 95% confident interval [CI]: 3.71–121.6; *p* = 0.0001) and its early administration (OR = 10.56, 95%CI: 1.90–58.5; *p* = 0.005) indicated significant higher risk of death in this study (Table 5).

**Table 5:** Risk factors for mortality of hemorrhagic shock following blunt trauma

	*Odds ratio (95% CI)*	*p value*
Activation of MTP	1.11 (0.021–58.49)	1.00
Aortic clamp including REBOA	0.55 (0.15–2.05)	0.51
Vasopressor use	21.32 (3.71–121.6)	0.0001
Vasopressor use < 1 h after admission	10.56 (1.90–58.5)	0.005

*CI* confidence interval, *MTP* massive transfusion protocol, *REBOA* resuscitative endovascular balloon occlusion of the aorta

## Discussion

The advantages and disadvantage of vasopressor administration or continuous volume resuscitation to improve the outcomes for these compromised patients with shock following blunt trauma are still unclear [12, 15, 22–25]. Thus, in this single-center retrospective observational study, we assessed the effects of vasopressor administration especially for the resuscitation of patients with severe blunt trauma. To evaluate the exact outcomes of vasopressor use, we included patients with backgrounds and injury severity that were not significantly different. Under these considerations, the present study found that high-dose use and early administration of vasopressors for the resuscitation of patients with severe blunt trauma had some potential relation to higher mortality. Although significantly more blood products were transfused in the surviving patients, this result potentially emphasizes the importance of the early administration of blood products and the provision of a continuous blood supply based on the 1:1:1 theory before administering high-dose vasopressors.

Globally, the recommendation of vasopressor administration is still quite uncertain. The European trauma care providers concluded that although vasopressors are frequently used, their level of recommendation is still controversial [26]. Even more, there are no reports of outcomes of long-term neurological function or quality of life so far.

For the resuscitation of patients with severe hemorrhagic shock following trauma, the first priority is the immediate control of bleeding [3]. However, how appropriate systemic perfusion especially for the cerebral circulation can be maintained or improved is also very important. Currently, although permissive hypotension especially for penetrating trauma is recommended [6, 14], the impact of permissive hypotension could be less beneficial or even cause harm in patients with blunt trauma at high risk for concomitant TBI [6–8]. Although we generally try to minimize the use of fluid resuscitation to avoid dilutional coagulopathy, there are certainly patients in whom we have difficulty in maintaining their blood pressure while performing bleeding control procedures. As is already known historically, excessive administration of external cellular fluids for resuscitation is completely disadvantageous and causes dilutional coagulopathy [2, 27, 28]. Thus, we limit the administered volume to < 1000 mL in hemorrhagic shock patients following trauma.

Although REBOA is now also one option available for the control of severe infra-diaphragm hemorrhage in trauma patients [29–31], as assessed globally, some outcomes are described in which patients undergoing REBOA placement had significantly more severe complications and higher mortality compared with patients not undergoing REBOA [32, 33]. Although the number of patients who had placed REBOA was too small to evaluate, REBOA placement had seemed no significant differences from the view point of resuscitation in our current study.

As this is the single-center retrospective study, the data have no institutional bias or differences of resuscitative strategies for shock trauma patients. But for the stronger evidence, prospective randomized surveys with larger numbers of patients are needed to evaluate the impact of vasopressor use in the early resuscitation phase and assess long-term outcomes of the patients with severe trauma.

### Limitations

The present study is a small, preliminary report from a single center, and the number of patients is too small to establish definitive conclusions. Thus, we need to plan further multi-institutional, prospective, randomized trials on the basis of this study to better assess the benefits and disadvantages of the administration of vasopressors for severe hemorrhagic shock in patients following blunt trauma.

## Conclusion

The present findings highlight that the administration of vasopressors and high-dose use in the resuscitation of hemorrhagic shock following severe blunt trauma are potentially associated with increased mortality. In the resuscitation of these patients, even if the transfused volume of blood products tends to be increased, the early termination of vasopressor therapy had better to be considered.

## Data Availability

The data generated and analyzed during this study are available from the authors on reasonable request.

## Abbreviations

TBI: Traumatic brain injury
MTP: Massive transfusion protocol
Ps: Probability of survival
TRISS: Trauma and injury severity score
FAST: Focused assessment with sonography for trauma
IQR: Interquartile range
ISS: Injury severity score
REBOA: Resuscitative endovascular balloon occlusion of the aorta
OR: Odds ratio
CI: Confident interval.
